# Prevalences of metabolic syndrome and its sex-specific association with socioeconomic status in rural China: a cross-sectional study

**DOI:** 10.1186/s12889-021-12074-z

**Published:** 2021-11-06

**Authors:** Xuhua Ying, Shuyue Yang, Songtao Li, Meifang Su, Na Wang, Yue Chen, Qingwu Jiang, Chaowei Fu

**Affiliations:** 1 Yuhuan City Center for Disease Control and Prevention, Yuhuan, Zhejiang Province China; 2 School of Public Health; Key Laboratory of Public Health Safety, NHC Key Laboratory of Health Technology Assessment, Fudan University, Shanghai, 200032 China; 3 School of Epidemiology and Public Health, Faculty of Medicine, University of Ottawa, Ottawa, Ontario Canada

**Keywords:** Metabolic syndrome, Socioeconomic status, Sex differences, East China

## Abstract

**Background:**

Few studies have reported the prevalence of metabolic syndrome (MS) and examined relationships between socioeconomic status and MS in rural China. This study aimed to evaluate the prevalence of MS and MS components as well as their associations with socioeconomic status among rural Chinese adults.

**Methods:**

A cross-sectional study of 26,836 participants aged 20 years and older was conducted from June to December 2012 in Yuhuan City, Zhejiang Province, China, which is located on Yuhuan Island. A multivariable logistic regression model was used to identify risk factors for MS and their possible interactions.

**Results:**

Among 26,836 subjects with an average age of 53.4 ± 14.0 years, 59% were female. The overall prevalence of MS was 20.5%, and there was a significant sex difference in the prevalence (15.1% for males vs. 24.2% for females, *P* < 0.001). Compared with males, females also showed a significantly higher proportion of most MS components. A significantly higher prevalence of MS was found among subjects who were elderly, had a lower income level, had a lower level of education, or were unemployed. Multiple significant interactions were observed between the prevalence of MS and sex, age or socioeconomic status (*P* < 0.001). The risk of MS increased significantly with age in females but not in males. Additionally, a lower income level and a lower level of education were significantly related to an increased risk only in females, and unemployed males had a higher risk of MS than unemployed females.

**Conclusions:**

The prevalence of MS and its components was relatively high in a rural island Chinese population with rapid urbanization, and sex-specific associations between socioeconomic factors and MS were found. Targeted preventive interventions should be developed and implemented to prevent and control MS among those with low socioeconomic status, especially females.

**Supplementary Information:**

The online version contains supplementary material available at 10.1186/s12889-021-12074-z.

## Background

Non-communicable chronic diseases (NCDs) are a major cause of mortality and an increasing socioeconomic burden globally [1–3]. It was exacerbated by rapid economic growth, aging population, and lifestyle changes [4]. Metabolic syndrome (MS), is characterized by clustering risk factors for NCDs, including central obesity, high blood pressure, raised hyperglycemia, and low high-density lipoprotein cholesterol [5]. The prevalence of MS has increased rapidly in both developed and developing countries and areas in recent years [6–10]. In China, this prevalence increased rapidly from 13.7% in 2005 to 33.9% in 2010 to 24.5% in 2016 [11–13]. Findings from previous studies have suggested that MS and its components are risk factors for atrial fibrillation, stroke, cardiovascular disease (CVD), diabetes mellitus (DM) and other NCDs [14, 15]. There were few studies about the status of MS and its possible influencing factors among island residents in rural China [16, 17]. Yuhuan County, located on Yuhuan Island, is one of the most developed areas in China and is experiencing rapid urbanization among rural residents; however, there is a relatively high prevalence of several chronic diseases due to high-salt and high-fat diets [18, 19].

Recent studies have shown that socioeconomic status (SES) is a strong predictor of morbidity and premature mortality both in developing and developed countries [20, 21]. Prior studies reported that lower SES was associated with a higher risk for NCDs such as type 2 diabetes and coronary heart disease, and the severity varied by sex [22, 23]. With the changing socioeconomic environment, few studies have examined the association between SES and MS in rural East China [24, 25]. This study aimed to estimate the prevalence of MS and the association between SES and MS in different sexes in a rural area with rapid urbanization in East China.

## Methods

### Study site and population

A cross-sectional study was conducted in 3 communities in Yuhuan City, Zhejiang Province, from June to December 2012 using cluster sampling. The studied communities included 1 street, 1 town and 1 township which were randomly selected from 3 streets, 6 towns and 2 townships of Yuhuan City respectively and all members were recruited into this study with a response rate of 78.4%. A total of 26,836 participants were included in this research, and the inclusion criteria were as follows: 1) 20 years and older; 2) local permanent resident without migration or travel plans (1 year or longer); 3) provided informed consent; and 4) able to complete the questionnaire and physical examination.

### Data collection and quality control

Face-to-face questionnaire interviews were conducted and anthropometric data were collected by trained local health professionals. The following demographic information data was collected with the questionnaire (seen in Supplementary file 1): age; sex; lifestyle information, including smoking history (non-smoker/former smoker/current smoker) [26], alcohol consumption (to drink at least once a week as yes) [27], and regular physical exercise (doing physical exercise at least 1 time a week, for at least 30 min each time, and feeling warm or sweaty) [28]; socioeconomic status, including years of education (< 9 years/≥9 years) [26], monthly household income (< 2000 RMB/≥2000 RMB), and occupation status (Manual worker/ Mental/ worker/ Unemployed/ Other) [29]; and health conditions, including disease history of hypertension, diabetes mellitus, stroke, cardiovascular disease or other [30]. Anthropometry data included height, weight, waist and hip circumference, and blood pressure.

Blood samples were collected after an 8-h fast and analyzed in a reference laboratory to assess blood biochemical indexes, including fasting plasma glucose (FPG), 2-h postprandial blood glucose (2hPG), total cholesterol (TC), low-density lipoprotein cholesterol (LDL-C), high-density lipoprotein cholesterol (HDL-C), and triglyceride (TG).

### Definition of metabolic syndrome

The diagnosis of metabolic syndrome was based on IDF criteria as follows:

Central obesity (defined as waist circumference ≥ 90 cm for males or ≥ 80 cm for females, or BMI > 30 kg/m^2^) plus any two or more of four additional factors:

1. Triglyceride levels ≥1.7 mmol/L (150 mg/dL);
2. High blood pressure: systolic blood pressure ≥ 130 mmHg or diastolic blood pressure ≥ 85 mmHg or treatment of previously diagnosed hypertension;
3. High-density lipoprotein cholesterol levels < 1.03 mmol/L (40 mg/dL) in males and 1.29 mmol/L (50 mg/dL) in females or specific treatment for lipid abnormalities;
4. Fasting plasma glucose levels ≥5.6 mmol/L (100 mg/dL) or previously diagnosed type 2 diabetes [31].

### Statistical analysis

All data were entered into Epidata 3.1 twice. Pearson’s chi-square test or Fisher’s exact test was applied for categorical variables. Student’s t-test or the Wilcoxon test was used for two-group comparisons of normally distributed and nonnormally distributed continuous variables, respectively. Logistic regression analysis was used to calculate crude odds ratios (cORs), adjusted ORs (aORs), and 95% confidential intervals (CIs) to estimate the potential risk factors as well as to explore the possible interactions between sex and socioeconomic factors. All analyses were conducted using SPSS 22.0, and a two-sided *P*-value of 0.05 or less was defined as significant, missing data were deleted.

## Results

### Basic characteristics of subjects (Table 1)

Data from 26,836 individuals were analyzed, including 59% females with an average age of 53.4 ± 14.0 years. Compared with male subjects, females were more likely to have lower levels of education, lower monthly household income levels, and unemployment. Males showed significantly higher proportions of smoking and alcohol consumption. Additionally, MS components, including hypertension, high FPG levels and high TG levels, were more common in males than in females, except for the low level of HDL-C. In addition, BMI and waist circumference were significantly higher in males than in females.

**Table 1:** Characteristics of subjects over sex in the rural Chinese population

Characteristics	Male, (%) (***n*** = 10,998)	Female, (%) (***n*** = 15,838)	Total, (%)	***P*** value^**e**^
Age group (years)				0.013
< 40	18.5	17.7	17.8	
40-	23.5	24.9	24.3	
50-	23.8	24.2	24.1	
60-	21.3	20.7	21.0	
≥70	13.4	12.4	12.8	
Education year (≥9)	12.4	10.8	11.5	< 0.001
Occupation status ^a^				< 0.001
Manual worker	32.7	18.2	24.1	
Mental worker	14.8	8.1	10.9	
Unemployed	28.4	62.5	48.5	
Others	24.0	11.2	16.5	
Regular physical exercise (Yes) ^b^	43.5	56.1	50.8	< 0.001
Monthly household income				< 0.001
< 2000 RMB	36.7	49.2	44.1	
≥2000 RMB	63.3	50.8	55.9	
Smoking				< 0.001
Non-smoker	52.9	99.5	80.4	
Former smoker	4.1	0.3	1.9	
Current smoker	43.0	0.2	17.7	
Alcohol consumption (Yes)	38.7	0.3	16.0	< 0.001
Hypertension (Yes) ^c^	59.2	49.2	53.3	< 0.001
Diabetes mellitus (Yes)	7.8	7.4	7.6	< 0.001
FPG (> 5.6 mmol/L) ^d^	28.3	23.6	25.5	< 0.001
TG (≥1.7 mmol/L)	33.3	23.5	27.5	< 0.001
HDL-C (< 1.03 mmol/L)	8.3	25.2	18.2	< 0.001
Age (years) (Mean ± SD)	53.6 ± 14.3	53.3 ± 13.8	53.4 ± 14.0	0.142
SBP, (mmHg), (Mean ± SD)	133.7 ± 18.9	129.9 ± 21.2	131.4 ± 20.3	< 0.001
DBP, (mmHg), (Mean ± SD)	79.4 ± 11.7	75.4 ± 10.9	77.0 ± 11.4	< 0.001
BMI, (kg/m2), (Mean ± SD)	24.1 ± 3.8	23.6 ± 3.7	23.8 ± 3.8	< 0.001
WC, (cm), (Mean ± SD)	83.0 ± 9.4	78.5 ± 9.6	80.3 ± 9.8	< 0.001
WHR (Mean ± SD)	0.90 ± 0.08	0.86 ± 0.09	0.88 ± 0.09	< 0.001

Notes: *FPG* Fasting plasma glucose, *TG* Triglyceride, *HDL-C* High-density lipoprotein cholesterol, *SBP* Systolic blood pressure, *DBP* Diastolic blood pressure, *BMI* Body mass index, *WC* Waist circumference, *WHR* Waist-to-hip ratio; ^a^:54 data loss; ^b^:2484 data loss; ^c^:7 data loss; ^d^:52 data loss.^e^:t or chi-square test

### Prevalence of MS and its influencing factors (Tables 2 & 3)

The overall prevalence of MS was 20.1%, and there was a significant sex difference in the prevalence between males and females (15.0% vs. 24.2%, *p* < 0.001). Additionally, participants who were elderly or unemployed, or had a lower monthly household income, or had a lower level of education, had a significantly higher prevalence of MS, and after the adjustment for covariates, there were significant interactions between sex and SES (*P* < 0.001). Males who were mental workers or unemployed had a higher risk of MS, and females with a lower monthly household income, lower education level, and unemployment had a higher risk of MS.

**Table 2:** Prevalence and possible risk factors of subjects for metabolic syndrome

Characteristics	Non-MS, (%) (***n*** = 20,908)	MS, (%) (***n*** = 5396) ^**a**^	***P*** value^**d**^	Crude OR (95%CI)	Adjusted OR (95%CI) ^**b**^
Sex			< 0.001		
Male	84.9	15.1		1.00	1.00
Female	75.8	24.2		1.79 (1.68, 1.91)	1.98 (1.79, 2.18)
Age group (years)			< 0.001		
< 40	93.1	6.9		1.00	1.00
40-	85.6	14.4		2.29 (2.00, 2.62)	2.22 (1.91, 2.57)
50-	77.0	23.0		4.05 (3.57, 4.60)	3.80 (3.29, 4.39)
60-	70.3	29.7		5.74 (5.05, 6.52)	5.40 (4.64, 6.28)
≥70	68.7	31.3		6.19 (5.41, 7.09)	5.86 (4.97, 6.90)
*P*-interaction ^c^					< 0.001
Monthly household income			< 0.001		
≥2000 RMB	83.7	16.3		1.00	1.00
< 2000 RMB	74.1	25.9		1.80 (1.70, 1.91)	1.07 (0.99, 1.15)
*P*-interaction ^c^					< 0.001
Education year			< 0.001		
≥9	89.2	10.8		1.00	1.00
< 9	78.2	21.8		2.29 (2.03, 2.58)	1.19 (1.04, 1.37)
*P*-interaction ^c^					< 0.001
Occupation status			< 0.001		
Manual worker	86.7	13.3		1.00	1.00
Mental worker	84.7	15.3		1.18 (1.04, 1.33)	1.64 (1.43, 1.89)
Unemployed	73.2	26.8		2.38 (2.19, 2.58)	1.43 (1.30, 1.58)
Others	84.0	16.0		1.24 (1.11, 1.38)	1.33 (1.18, 1.50)
*P*-interaction ^c^					< 0.001
Smoking			< 0.001		
Non-smoker	78.3	21.7		1.00	1.00
Former smoker	85.1	14.9		0.63 (0.49, 0.81)	0.91 (0.69, 1.19)
Current smoker	84.3	15.7		0.67 (0.62, 0.73)	1.15 (1.02, 1.29)
*P*-interaction ^c^					0.205
Alcohol consumption			< 0.001		
No	78.8	21.2		1.00	1.00
Yes	83.3	16.7		0.74 (0.68, 0.81)	1.28 (1.14, 1.45)
*P*-interaction ^c^					0.533

Notes: ^a^: 532 data loss. ^b^: Adjusted for sex, age group, monthly household income, education year, occupation status, regular physical exercise, smoking, alcohol consumption; ^c^: Interactions between sex and socioeconomic factors, including age group, monthly household income, education year, unemployed. ^d^: Chi-square test

**Table 3:** Different risk-factors of subjects by sex for metabolic syndrome

Characteristics	OR (95% CI) ^**a**^	Male (***n*** = 10,998)		Female (***n*** = 15,838)	
		MS, (%) ^**b**^	Adjusted OR (95%CI)	MS, (%) ^**c**^	Adjusted OR (95%CI)
Monthly household income					
≥ 2000 RMB	1.00	15.3	1.00	17.1	1.00
< 2000 RMB	2.48 (2.15, 2.87)	14.9	0.83 (0.73, 0.95)	31.6	1.20 (1.10, 1.32)
Education year					
≥ 9	1.00	13.8	1.00	8.4	1.00
< 9	3.48 (2.67, 4.53)	15.3	0.90 (0.75, 1.09)	26.1	1.56 (1.27, 1.93)
Occupation status					
Manual worker	1.00	11.5	1.00	15.6	1.00
Mental worker	0.44 (0.33, 0.58)	17.1	1.70 (1.42, 2.04)	13.1	1.17 (0.94, 1.46)
Unemployed	1.28 (1.05, 1.56)	17.9	1.70 (1.45, 2.00)	29.5	1.20 (1.05, 1.38)
Others	0.69 (0.54, 0.88)	15.7	1.37 (1.16, 1.61)	16.4	1.08 (0.89, 1.31)

Notes: Adjusted for age group, monthly household income, education year, occupation status, smoking, alcohol consumption, regular physical exercise. ^a^: This column represents the OR (95%CI) for the interaction; ^b^:255 data loss; ^c^:277data loss

### Sex-specific prevalence of MS components (Table 4)

The prevalence of MS components (except HDL-C) among females increased with age, but only the prevalence of hypertension among males increased with age. It was revealed that females had higher prevalences of most MS components, especially HDL-C, than males in all age groups, whereas there were higher proportions of triglyceride levels, high blood pressure, and FPG levels among males under the age of 70 (Supplementary file 2: Supplementary Fig. 1). As presented in Table 4, females had a higher risk of HDL-C and central obesity components, while males had higher triglyceride levels, higher blood pressure, and higher FPG levels after the adjustment for demographic and other MS components.

**Table 4:** Associations between sex and metabolic syndrome components among subjects

MS components	Crude OR (95%CI)	***P*** value	Adjusted OR (95%CI)	***P*** value
Raised triglycerides ^a^	0.61 (0.58, 0.65)	< 0.001	0.45 (0.41, 0.49)	< 0.001
High blood pressure ^b^	0.67 (0.64, 0.70)	< 0.001	0.57 (0.52, 0.62)	< 0.001
High FPG ^c^	0.79 (0.75, 0.83)	< 0.001	0.72 (0.66, 0.78)	< 0.001
Low HDL-C ^d^	3.74 (3.46, 4.04)	< 0.001	4.57 (4.01, 5.22)	< 0.001
Central obesity ^e^	2.48 (2.35, 2.62)	< 0.001	2.87 (2.63, 3.13)	< 0.001

Notes: Male were taken as the reference group. Fixed covariates: age, education level, monthly household income, occupation status, regular physical exercise, smoking, alcohol drinking; ^a^: Adjusted for fixed covariates + BMI, HBP, HDL-C, FPG, Central obesity; ^b^: Adjusted for fixed covariates + BMI, TG, HDL-C, FPG, Central obesity; ^c^: Adjusted for fixed covariates + BMI, HBP, TG, HDL-C, Central obesity; ^d^: Adjusted for fixed covariates + BMI, HBP, TG, FPG, Central obesity; ^e^: Adjusted for fixed covariates+ HBP, TG, HDL-C, FPG

## Discussion

In this study, we found a high prevalence of MS of 20.1% among 26,836 participants in Yuhuan County, a coastal area located in developed East China, which was similar to previous studies ranging from 22.0 to 25.3%, and a higher prevalence for females was observed, which was consistent with previous studies [15, 32, 33]. Compared with the Northwest or other developing areas in China, MS was more prevalent in East China [34, 35]. The prevalence of MS increased with age in females, while in males, it first increased and then decreased, which was similar to the findings of other studies [36]. Furthermore, the prevalence in females was higher than that in males among individuals over the age of 50 years old and among those over the age of 60 years old, which may be associated with menopausal estrogen deficiency [37, 38].

The following shuld be merged into the above paragraph, which is a comment and should be removed. Another explanation was that the sex hormones affecting skeletal muscle mass were decreased in elderly and female subjects, which resulted in lower muscle mass and a higher risk of NCDs [39, 40]. Such an increase for women may also result from similar age-related trends in all MS components except HDL-C (Supplementary file 2: Supplementary Fig. 1).

Another finding of this study was that significant interactions on MS between sex and SES were observed among individuals. In this study, females with a lower SES (especially lower income and lower education level) showed a higher risk for MS, which was consistent with prior studies [41–44]. This finding could be explained by the following potential reasons. On one hand, participants with a lower SES were more likely to be exposed to unhealthy behaviors, psychological distress, lower life expectancy, lower access to health care, lower awareness of disease prevention and control, and a series of NCDs, such as obesity, hypertension, and diabetes mellites, which are commonly associated with MS [21, 41–43]. In another hand, compared with females, lower income was shown to be a protective factor for MS among males, which may be due to males being more likely to have a manual occupation with reduced risk for MS [44, 45]. Nam’s study reported that office workers had a higher risk of MS than manual workers, which was consistent with the present study [46].

### Limitations and strengths

This study was carried out in the rural Chinese community population with rapid urbanization, and the findings should be helpful to understand the status of MS in other similar areas of China or other countries. Of course, some limitations should be discussed. First, this study population was from Zhejiang Province, one of the developed areas in China, where the socioeconomic and lifestyle factors may be different than those in other rural areas in China. For example, the per capita Gross domestic product (GDP) of Yuhuan was 88,421 RMB in 2012, which was higher than the average level of China [47, 48]. Second, anthropometric and demographic information was collected at a single point, which may lead to data inaccuracy, and it was less feasible for the present study to only use IDF criteria as a definition without using other new definitions. Third, as a cross-sectional study, it is not possible to draw causal conclusions. Fourth, demographic information was self-reported and collected by questionnaires, which may lead to report bias. Fifth, male and female may come from the same family and share the same monthly household income number which could bias the findings. In addition, it was noted that some potential confounders such as diet factors were not controlled in this study. A prospective cohort study should be conducted in the future to confirm the causal association between SES factors and the incident of MS, especially regarding sex differences.

## Conclusions

Our study indicated the different distribution of MS prevalence and MS components in males and females as well as sex differences in the association between SES and MS. This study also updated the prevalence data in the Yuhuan rural area in Zhejiang Province in China. Risk factors for MS were identified to provide a reference for the prevention and management of this disease. More interventions and policies regarding the risk factors for MS need to be applied for people with lower SES, especially in females, to increase access to health care and to reduce health inequalities.

## Supplementary Information

**Additional file 1: Supplementary file 1.** Baseline Questionnaire of Yuhuan Population Health Cohort Study.

**Additional file 2: Supplementary file 2.** Supplementary Figure 1. Prevalence of metabolic syndrome and its components in different age groups over sex. Notes: TG: Triglyceride; BP: Blood pressure; FPG: Fasting plasma glucose; HDL-C: High-density lipoprotein cholesterol, A: Total; B: Male group; C: Female group.

## Data Availability

The datasets generated and/or analysed during the current study are not publicly available owing to local legislation and the written consent forms of participants but are available from the corresponding author on reasonable request.

## Abbreviations

MS: Metabolic syndrome
IDF: International Diabetes Federation
ORs: Odds ratios
CIs: 95% confidence intervals
BMI: Body mass index
NCDs: Non-communicable chronic disease
SES: Socioeconomic status
AF: Atrial fibrillation
CVD: Cardiovascular disease
DM: Diabetes mellitus
FPG: Fasting plasma glucose
2hPG: 2-h postprandial blood glucose
TC: Total cholesterol
LDL-C: Low-density lipoprotein cholesterol
HDL-C: High-density lipoprotein cholesterol
TG: Triglyceride
SBP: Systolic blood pressure
DBP: Diastolic blood pressure
HBP: High blood pressure
