# Supplementary material for: Prevalences of metabolic syndrome and its sex-specific association with socioeconomic status in rural China: a cross-sectional study
Source: BMC Public Health. 2021 Nov 6;21:2033. doi: 10.1186/s12889-021-12074-z (PMC8572445; doi:10.1186/s12889-021-12074-z)
Supplement: Supplementary file 1 — Additional file 1: Supplementary file 1. Baseline Questionnaire of Yuhuan Population Health Cohort Study. [file 12889_2021_12074_MOESM1_ESM.docx]

Baseline Questionnaire of Yuhuan Population Health Cohort Study No：__________

| **1. Demographic information** | | | | | | | | | | | | | | | | | | | | | |
| --- | --- | --- | --- | --- | --- | --- | --- | --- | --- | --- | --- | --- | --- | --- | --- | --- | --- | --- | --- | --- | --- |
| Name | |  | | | | | Sex | | | 1.Male 2.Female | | | | | | Telephone | | |  | | |
| ID | |  | | | | | | | | Address | | | | | |  | | | | | |
| Education level | | 1.Primary school and below 2. Junior high school 3.high school 4.college and above | | | | | | | | | | | | | Monthly household income per capita in one recent year | | | | | | _____RMB |
| Marital status | | 1. unmarried 2. Married 3. Divorced 4. widowed | | | | | | | | | | | | | Han nationality | | | | | | 1.Yes 2.No |
| Occupation | | Employed：1.Cilvil servant 2. Peasant 3. Worker 4. Merchant 5. Enterprise manager 6. Private entrepreneur 7. Employees of public institutions 8. Professional and technical staff  9. Other  Unemployed：1.Unemployment 2. Household work 3. Retirement | | | | | | | | | | | | | | | | | | | |
| **2. Whether have diabetes** 1. Yes 2. No (Jump to section4) | | | | | | | | | | | | | | | | | | | | | |
| Diagnosed date | |  | | | Diagnosed hospital level | | | | | | | 1. Provincial 2. Municipal 3. County 4. Community 5. Unknown | | | | | | | | | |
| Diagnosed way | | 1.Health records 2.Physical examination 3.Hospital 4.Self-report of polydipsia, polyuria, polyphagia, weight loss 5.Other：__________ | | | | | | | | | | | | | | | | | | | |
| Latest fasting blood glucose glucose | | | | | | mmol/l | | | | | Latest 2 hours OGGT blood glucose | | | | | | | | | mmol/l | |
| Latest hemoglobin a1c | | | | | | | | | | | % | | | | | | | | | | |
| **3. Whether have any hypoglycemic treatment** 1. Yes 2. No (Jump to section 4) | | | | | | | | | | | | | | | | | | | | | |
| Drug name | Each dose | | | Drug taking method | | | | | | | | | Times/day | | | | | Duration of medicine(year) | | | |
|  |  | | | 1.Oral 2.Injection | | | | | | | | |  | | | | |  | | | |
|  |  | | | 1.Oral 2.Injection | | | | | | | | |  | | | | |  | | | |
|  |  | | | 1.Oral 2.Injection | | | | | | | | |  | | | | |  | | | |
|  |  | | | 1.Oral 2.Injection | | | | | | | | |  | | | | |  | | | |
| **4. Symptoms of Diabetes** | | | | | | | | | | | | | | | | | | | | | |
| Polydipsia | | | | | | | | 1.Yes 2.No | | | | Polyphagia | | | | | | | | | 1.Yes 2.No |
| Polyuria | | | | | | | | 1.Yes 2.No | | | | Weight loss | | | | | | | | | 1.Yes 2.No |
| Skin infection or tuberculosis | | | | | | | | 1.Yes 2.No | | | | Blurring of vision | | | | | | | | | 1.Yes 2.No |
| Edema of lower limbs | | | | | | | | 1.Yes 2.No | | | | Hands and feet numbness | | | | | | | | | 1.Yes 2.No |
| **5. Combined diseases** (**Multiple choice**) 1.Yes 2.No (Jump to section 6) | | | | | | | | | | | | | | | | | | | | | |
| Disease name | | | Diagnosed  date | | | | | | Disease name | | | | | Diagnosed date | | | Disease name | | | | Diagnosed  date |
| Hypertension | | |  | | | | | | Myocardial  infarction | | | | |  | | | Coronary heart disease | | | |  |
| Stroke | | |  | | | | | | Retinopathy | | | | |  | | | hyperlipidemia | | | |  |
| Diabetes  nephropathy | | |  | | | | | | Diabetic foot | | | | |  | | | Other：______ | | | |  |
| **6. Current lifestyle** | | | | | | | | | | | | | | | | | | | | | |
| Physical activities (**Multiple choice**) (Definition：Doing physical exercise at least 1 time a week, for at least 30 minutes each time, and feeling warm or sweaty) | | | | | | | | | | | | | | | | | | | | | |
| 1.No/Barely  2.Occupational physical activity, main ways：__________,_____times/week，______minutes/time  3.household work，main way：__________,_____times/week，______ minutes/time  4.Physical exercise，main way：__________,_____ times/week，______ minutes/time | | | | | | | | | | | | | | | | | | | | | |
| Smoking (Definition：At least 1 cigarette per day for up to 6 months) | | | | | | | | | | | | | | | | | | | | | |
| 1.No 2.Smoking cessation, average cigarette/day 3.Yes, average cigarette/day | | | | | | | | | | | | | | | | | | | | | |
| Passive smoking (Definition：Someone smokes near you or in the same room) | | | | | | | | | | | | | | | | | | | | | |
| 1.No 2.Occasionally 3.Often，average cigarette/day， minutes/day | | | | | | | | | | | | | | | | | | | | | |
| Alcohol consumption (Definition：At least 1 drink per week) | | | | | | | | | | | | | | | | | | | | | |
| 1.No 2.Yes，average white wine ml /day，yellow wine ml/day，red wine ml/day，beer ml/day | | | | | | | | | | | | | | | | | | | | | |
| **7. Risk factors** | | | | | | | | | | | | | | | | | | | | | |
| Birth weight | | | | | | ________g | | | | | Parents, child or sibling with diabetes | | | | | | | | | | 1.Yes 2.No |
| impaired glucose regulation | | | | | | 1.Yes 2.No | | | | | History of glucocorticoid-induced diabetes | | | | | | | | | | 1.Yes 2.No |
| History of gestational diabetes | | | | | | 1.Yes 2.No | | | | | History of delivery of macrosomia (birth weight ≥4kg) | | | | | | | | | | 1.Yes 2.No |
