# Supplementary material for: Prevalences of metabolic syndrome and its sex-specific association with socioeconomic status in rural China: a cross-sectional study
Source: BMC Public Health. 2021 Nov 6;21:2033. doi: 10.1186/s12889-021-12074-z (PMC8572445; doi:10.1186/s12889-021-12074-z)
Supplement: Supplementary file 2 — Additional file 2: Supplementary file 2. Supplementary Figure 1. Prevalence of metabolic syndrome and its components in different age groups over sex. Notes: TG: Triglyceride; BP: Blood pressure; FPG: Fasting plasma glucose; HDL-C: High-density lipoprotein cholesterol, A: Total; B: Male group; C: Female group. [file 12889_2021_12074_MOESM2_ESM.docx]

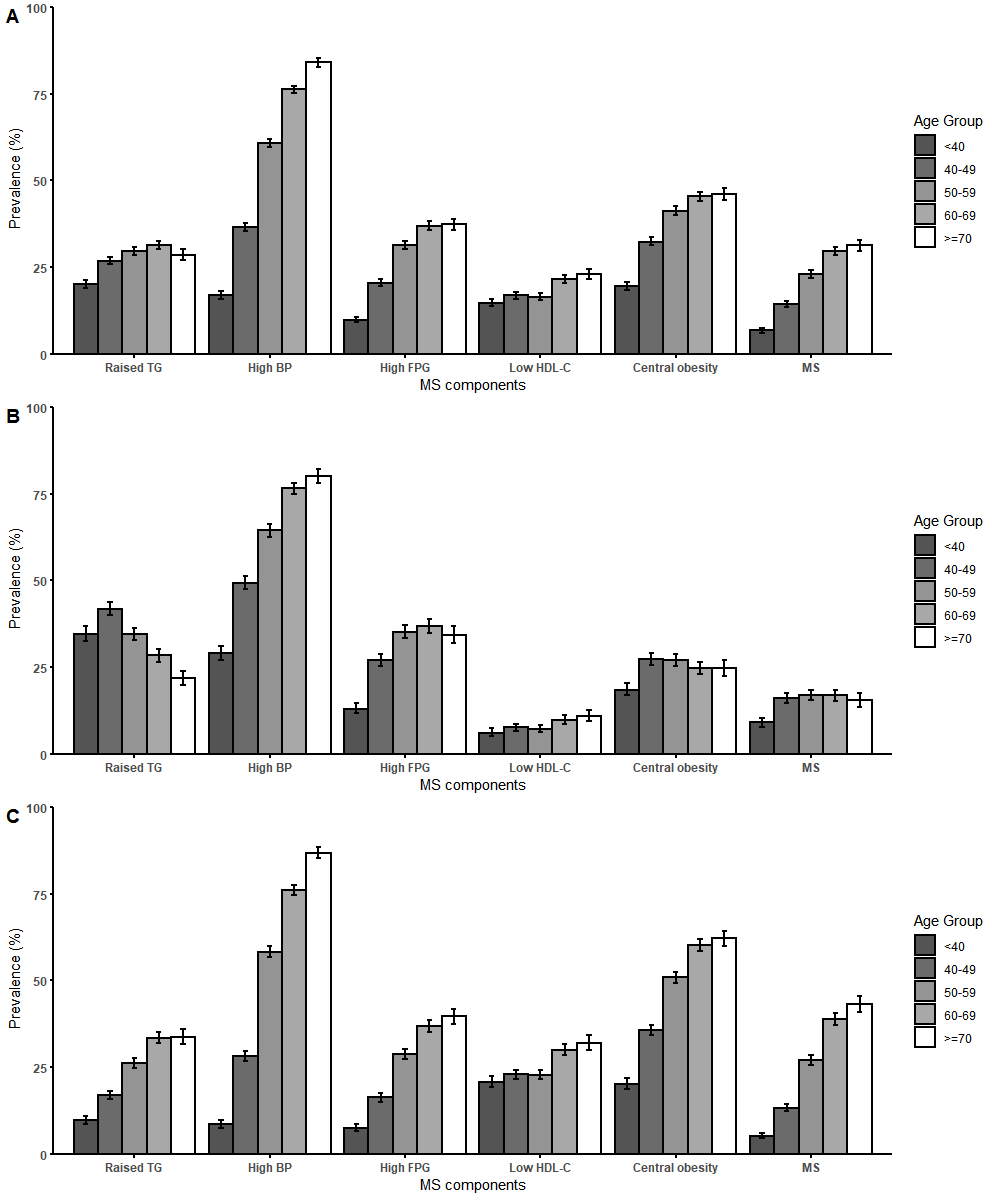
 Supplementary figure 1 Prevalence of metabolic syndrome and its components in different age groups over sex

Notes: TG: Triglyceride; BP: Blood pressure; FPG: Fasting plasma glucose; HDL-C: High-density lipoprotein cholesterol, A: Total; B: Male group; C: Female group.
